# Characterizing intubation practices in response to the COVID-19 pandemic: a survey of the Canadian COVID-19 Emergency Department Rapid Response Network (CCEDRRN) sites

**DOI:** 10.1186/s12873-023-00911-w

**Published:** 2023-11-24

**Authors:** Muzeen Ismath, Holly Black, Carmen Hrymak, Rhonda J. Rosychuk, Patrick Archambault, Patrick T. Fok, Thomas Audet, Brenden Dufault, Corinne Hohl, Murdoch Leeies

**Affiliations:** 1 Department of Emergency Medicine, University of Manitoba, Winnipeg, MB Canada; 2 Department of Pediatrics, University of Alberta, Edmonton, AB Canada; 3 Department of Family Medicine and Emergency Medicine, Department of Anesthesiology and Intensive Care, Université Laval, Québec, QC Canada; 4 Department of Emergency Medicine, Dalhousie University, Halifax, NS Canada; 5 Department of Internal Medicine, Université Laval, Québec, QC Canada; 6 George & Fay Yee Centre for Healthcare Innovation, Winnipeg, MB Canada; 7 Department of Community Health Sciences, Rady Faculty of Health Sciences, University of Manitoba, Winnipeg, MB Canada; 8 Deparment of Emergency Medicine, University of British Columbia, Vancouver, BC Canada; 9 Rady Faculty of Health Sciences, Section of Critical Care Medicine, University of Manitoba, Winnipeg, MB Canada

**Keywords:** SARS-CoV-2, COVID-19, COVID, Emergency airway management, Emergency intubation, Clinical simulation, In situ simulation

## Abstract

**Objective:**

The risk of occupational exposure during endotracheal intubation has required the global Emergency Medicine (EM), Anesthesia, and Critical Care communities to institute new COVID- protected intubation guidelines, checklists, and protocols. This survey aimed to deepen the understanding of the changes in intubation practices across Canada by evaluating the pre-COVID-19, early-COVID-19, and present-day periods, elucidating facilitators and barriers to implementation, and understanding provider impressions of the effectiveness and safety of the changes made.

**Methods:**

We conducted an electronic, self-administered, cross-sectional survey of EM physician site leads within the Canadian COVID-19 Emergency Department Rapid Response Network (CCEDRRN) to characterize and compare airway management practices in the pre-COVID-19, early-COVID-19, and present-day periods. Ethics approval for this study was obtained from the University of Manitoba Health Research Ethics Board. The electronic platform SurveyMonkey (www.surveymonkey.com) was used to collect and store survey tool responses. Categorical item responses, including the primary outcome, are reported as numbers and proportions. Variations in intubation practices over time were evaluated through mixed-effects logistic regression models.

**Results:**

Invitations were sent to 33 emergency department (ED) physician site leads in the CCEDRRN. We collected 27 survey responses, 4 were excluded, and 23 analysed. Responses were collected in English (87%) and French (13%), from across Canada and included mainly physicians practicing in mainly Academic and tertiary sites (83%). All respondents reported that the intubation protocols used in their EDs changed in response to the COVID-19 pandemic (100%, n = 23, 95% CI 0.86-1.00).

**Conclusions:**

This study provides a novel summary of changes to airway management practices in response to the evolving COVID-19 pandemic in Canada. Information from this study could help inform a consensus on safe and effective emergent intubation of persons with communicable respiratory infections in the future.

**Supplementary Information:**

The online version contains supplementary material available at 10.1186/s12873-023-00911-w.

## Introduction

The risk of occupational exposure to Severe Acute Respiratory Syndrome Coronavirus 2 (SARS-CoV-2) during endotracheal intubation [1] has required global emergency medicine (EM) communities to institute new Coronavirus infectious disease (COVID)-protected intubation protocols and procedures [2–10]. Given the necessity for rapid implementation, many protocols are based on expert opinion, local experience, and limited observational data, but consensus on the optimal approach does not exist [11–13]. Nevertheless, high first-pass success rates and low rates of adverse events have been reported in patients with confirmed or suspected SARS-CoV-2 across Canada [14]. Our survey aimed to evaluate whether emergency medicine intubation protocols changed in response to the COVID-19 pandemic. We also sought to identify facilitators and barriers to implementation, and to understand provider perspectives on the effectiveness and safety of the changes made.

## Methods

### Study design

We conducted an electronic, self-administered, cross-sectional survey to characterize and compare airway management practices in the pre-COVID-19 (before March 11, 2020), early-COVID-19, and present-day periods (January-April, 2022).

Survey domains included variations in intubation practices, timing of intubation, variations in peri-intubation management strategies, intubation team structures, occupational safety, barriers or facilitators to implementing novel intubating processes, and respondent impressions of effectiveness and patient safety regarding intubation protocol changes. We developed our questionnaire and survey administration strategy adhering to the Burns [15] and Dillman methods [16]. Items were generated iteratively through discussion with content experts and collaborators using an Ishikawa causal effect framework to detail and organize components of the complex airway management process [17]. Items were grouped by domain, and collaborators generated items until thematic saturation was achieved. Items were reduced iteratively via a modified Delphi process. The questionnaire was pilot tested with both collaborators and a sample of EM resident physicians to assess the survey tool’s face and content validity, comprehensiveness, and clarity. Pilot participants recorded the time to complete the survey. The questionnaire was translated and back-translated from English to French. Finally, the SurveyMonkey platform (www.surveymonkey.com) was used to create an electronic questionnaire.

### Study population

We surveyed EM physician site leads within the Canadian COVID-19 Emergency Department Rapid Response Network (CCEDRRN) which harmonized data collection from patients with suspected and confirmed COVID-19 in a subset of EDs across Canada from March 1, 2020 onward [18]. CCEDRRN is currently the third largest COVID registry listed by the WHO (CCEDRRN.com). At the time of this survey CCEDRRN included 33 active site leads representing 39 EDs. CCEDRRN site leads in each participating centre received a personalized introductory email communicating the survey objectives with an invitation to participate. This letter included details of survey endorsements, a $5 coffee gift card unconditional incentive, and a link to the survey website. Electronic reminders were sent to non-respondents at weeks 2, 4, and 6. Survey completion was voluntary, and respondent identifying information was not linked to survey responses. Informed consent was obtained from all participants via a consent disclosure statement embedded in the invitation.

### Outcome measures

The primary outcome was the proportion of respondents reporting a change to their Emergency Department (ED) site intubation practices in response to the COVID-19 pandemic. Secondary outcomes included specific differences between pre-COVID-19, early-COVID-19, and present-day periods in intubation team structures, equipment, medications, clinical factors, processes, and occupational safety related to emergent intubation. Additional outcomes included the perceived efficacy and patient safety of intubation practices, changes in quality assurance practice, as well as barriers and facilitators of the rapid implementation of novel intubation practices.

### Data collection

The electronic platform SurveyMonkey (www.surveymonkey.com) was used to collect and store questionnaire responses. The questionnaire is appended (Appendix 1). Data collection occurred from January-April, 2022.

### Data analysis

Categorical item responses, including the primary outcome, are reported as frequencies and proportions. We report our primary outcome as a binary proportion with a 95% confidence interval calculated via the Wilson method.

### Sample size

A response rate of 66% was targeted. Evidence-based survey science strategies have achieved response rates of 54–71% in similar sample populations [19–21]. There were 33 ED physician site leads in the CCEDRRN when we conducted our survey, with some representing multiple sites. Assuming a population of N = 33, a sample size of n = 20 (66%) offered an ability to evaluate proportions with a 95% confidence level margin of error of 5% [17].

## Results

**Fig. 1:**
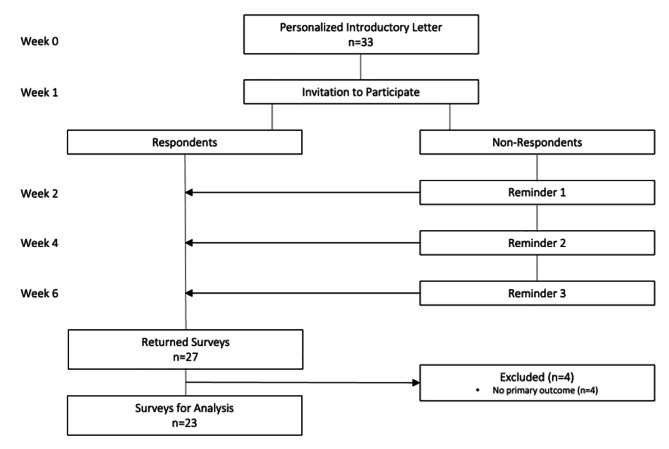
Survey response flow diagram

Invitations were sent to 33 ED physician site leads in the CCEDRRN. We received 27 survey responses, representing a response rate of 82%. Of the 27 returned surveys, 4 responses were excluded based on missing primary outcome variables, leaving 23 surveys for analysis (Fig. 1).

### Baseline characteristics

Respondents completed our questionnaire in English (87%) and French (13%). Most respondents were in practice 5 years or less. Respondents were geographically located across Canada. Respondents primarily practiced in academic/university (83%) and/or tertiary (35%) EDs, with fewer respondents practicing in community centres (17%). All respondents practiced at a centre where mechanically ventilated patients are admitted on-site (100%). Most respondents intubated 1–5 patients with confirmed or suspected COVID-19 (Table 1).

**Table 1:** Baseline Characteristics

	Responses (N = 23)
*Response Language, n (%)*	
English	20 (87)
French	3 (13)
*Duration of practice, n (%)*	
0–5 years	8 (35)
6–10 years	6 (26)
11–15 years	3 (13)
> 15 years	6 (26)
*Province/Territory of practice, n (%)*	
British Columbia	5 (22)
Alberta	3 (13)
Saskatchewan	1 (4)
Manitoba	3 (13)
Ontario	3 (13)
Quebec	4 (17)
Nova Scotia	3 (13)
New Brunswick	1 (4)
Prince Edward Island	0 (0)
Newfoundland/Labrador	0 (0)
Yukon	0 (0)
Northwest Territories	0 (0)
Nunavut	0 (0)
Practice setting*, n (%)	
Academic/University centre	19 (83)
Tertiary centre	8 (35)
Community centre	4 (17)
Admission practice, n (%)	
Mechanically ventilated patients are admitted on site	23 (100)
Mechanically ventilated patients are transferred off-site for admission	0 (0)
*Number of patients with confirmed or suspected COVID-19 the respondent has personally intubated during the COVID-19 pandemic, n (%)*	
0 patients	2 (9)
1–5 patients	8 (35)
6–10 patients	7 (30)
11–20 patients	4 (17)
21–30 patients	1 (4)
>30 patients	1 (4)

*Respondents could select multiple options so variable will not sum to 100%

### Primary outcome

Respondents reported that the intubation protocols used in their EDs changed in response to the COVID-19 pandemic (100%, n = 23, 95% CI 0.86-1.00).

### Secondary outcomes

#### Team structures

**Fig. 2:**
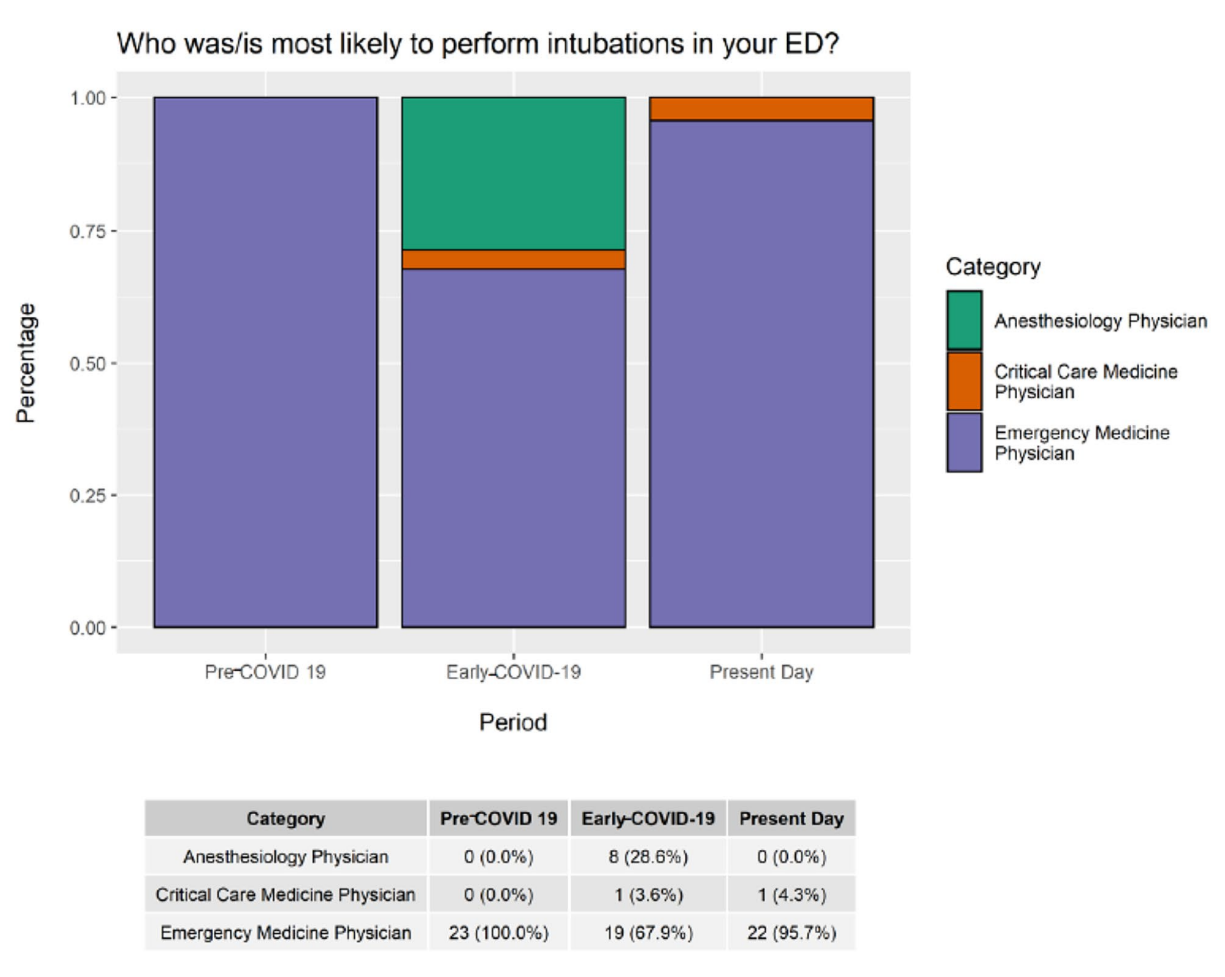
Intubator role over time

In the pre-COVID-19 era, all respondents reported EM physicians were most likely to perform intubations in their ED (100%, n = 23). In the early-COVID-19 period, an increase in ED intubations by anaesthesiologists and critical care physicians occurred (Anaesthesia 35% n = 8, Critical Care 4%, n = 1). This distribution shifted again in the present-day period, with sites reporting EM physicians being most likely to intubate (EM 96%, n = 22 vs. Critical Care 4%, n = 1 vs. Anaesthesia 0%, n = 0) (Fig. 2).

Trainee involvement in intubation varied over time. Junior medical trainees were more likely to be excluded from performing supervised intubation during the early-COVID-19 period (pre-COVID-19, 91% allowed junior residents, 70% allowed medical students vs. early-COVID-19 9% of sites allowed junior residents, 4% allowed medical students) (Appendix Fig. 1). The COVID-19 pandemic also saw the introduction of dedicated hospital-wide (43%) or dedicated in-ED (4%) intubation teams (Appendix Fig. 2).

#### Equipment

**Fig. 3:**
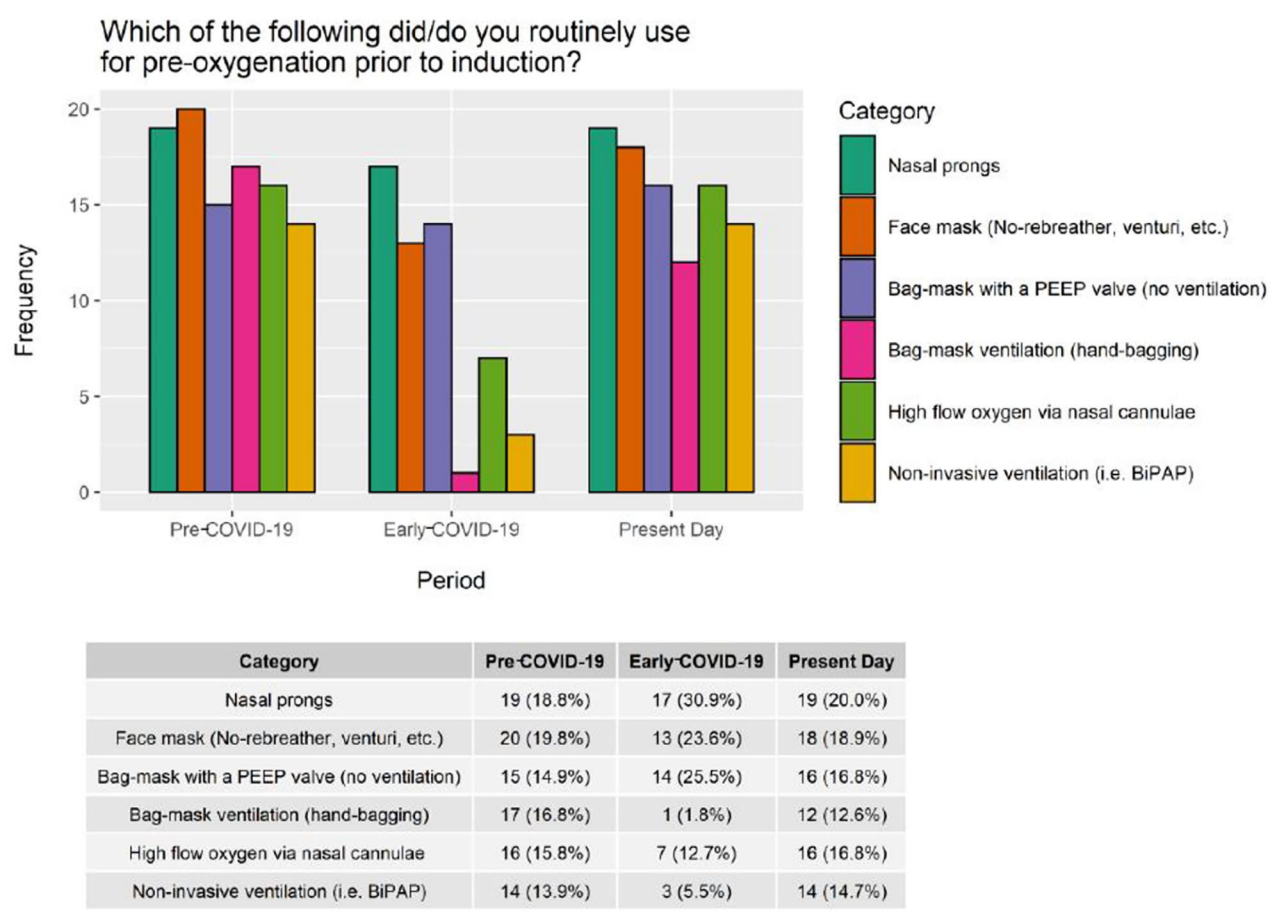
Variation in pre-oxygenation modalities over time PEEP: positive end-expiratory pressure, BiPAP: Bi-level positive airway pressure

**Fig. 4:**
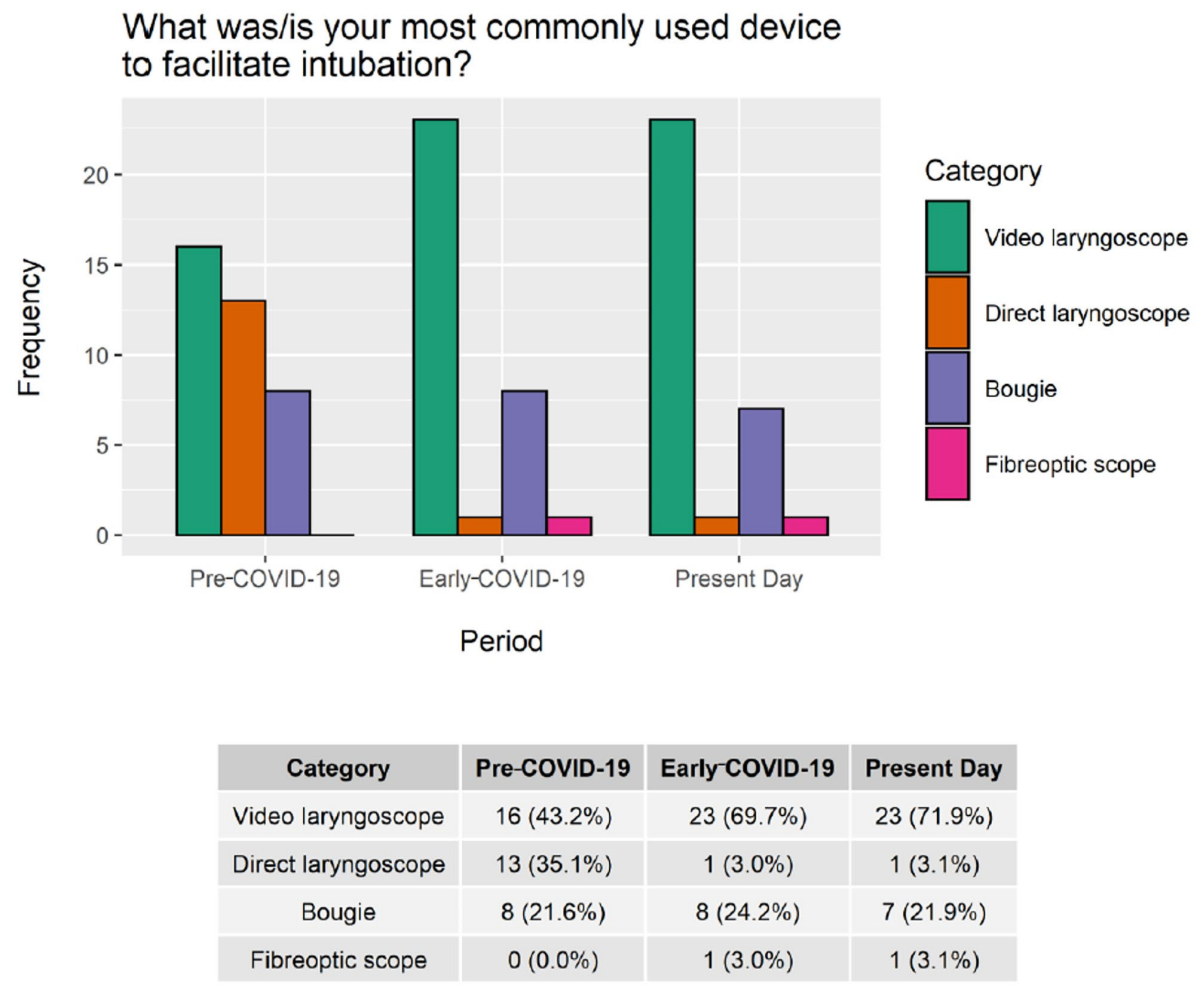
Variation in laryngoscopy/bougie use over time Respondents were able to select multiple options

Respondents reported bimodal use of oxygen delivery devices for pre-oxygenation, with higher rates of bag-mask ventilation, high-flow nasal cannulae, and non-invasive ventilation in the pre-COVID-19 and present-day periods, followed by decreased use during the early-COVID-19 period. Variations in bag-mask with a positive end-expiratory pressure (PEEP) valve (but no ventilation), face mask, and nasal prong use was to a smaller degree (Fig. 3). Additionally, we noted an increase in video laryngoscopy (VL) use in Canada during the early-COVID-19 period, which continued into the present-day (Fig. 4). During the pandemic, nearly a third of practitioners (30%, n = 7) adopted intubation bags/boxes but over half (57%, n = 4) subsequently abandoned them in the present-day. Auscultation, used to confirm endotracheal tube placement, decreased in the early COVID-19 period. Differences in rescue modalities in the event of a failed intubation attempt were not statistically significant (Appendix Fig. 3).

#### Medications

Induction medication selection varied over time, with ketamine being the most frequently reported induction agent in the pre-COVID-19 (87%, n = 20), early-COVID-19 (91%, n = 21), and present-day periods (91%, n = 21) (Appendix Fig. 4). Paralytics, used to facilitate intubation, were common during all periods (91%, n = 21 pre-COVID-19 vs. 100%, n = 23 in both early-COVID-19 and present-day periods).

#### Clinical factors

**Fig. 5:**
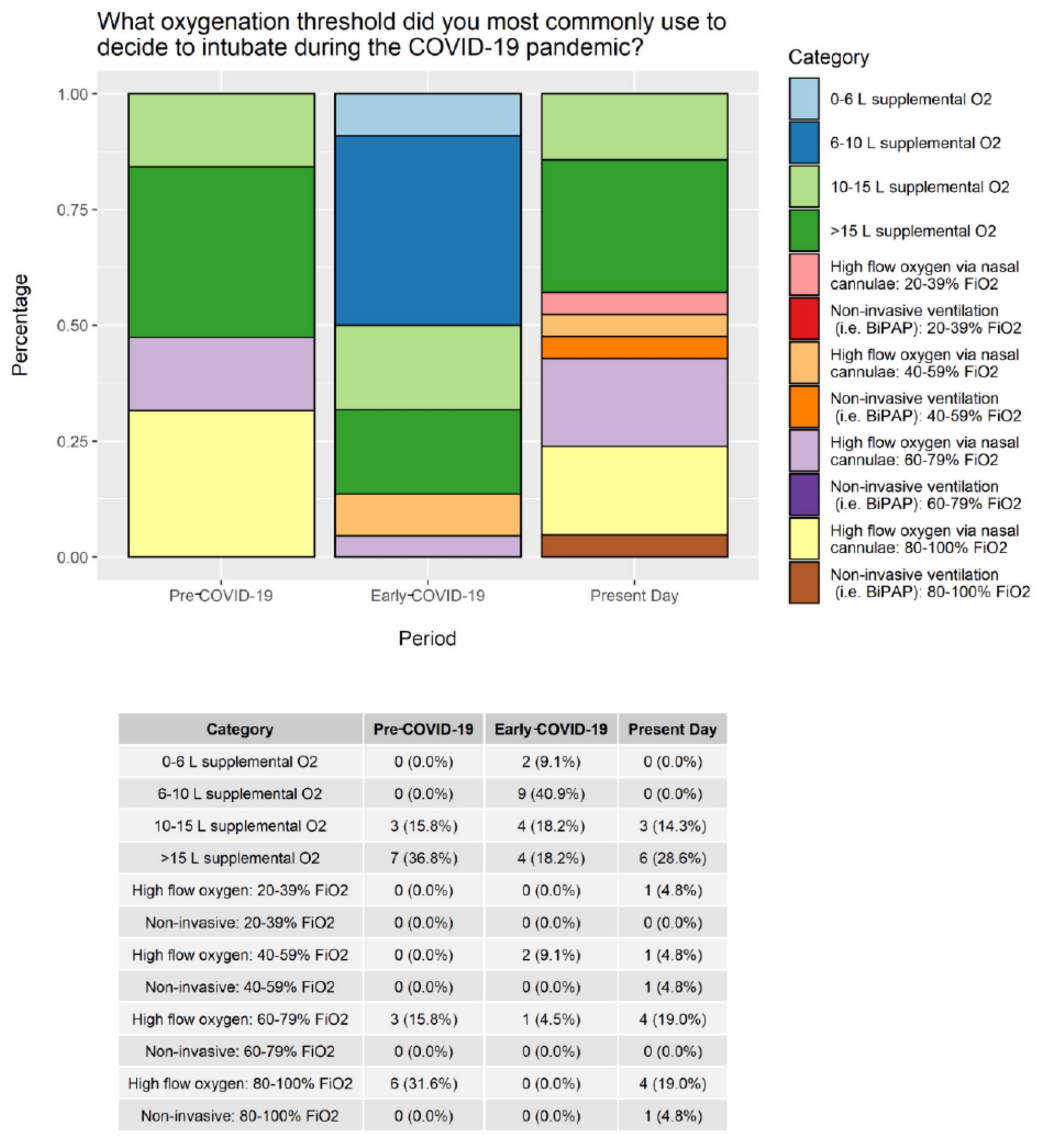
Oxygenation thresholds for intubation over time O2: oxygen; FiO2: fraction of inspired oxygen; BiPAP: bi-level positive airway pressure

Respondents reported changes to their clinical threshold for intubation during COVID-19, being more likely to intubate patients at lower oxygen requirement and/or work of breathing thresholds early on in the pandemic (78%, n = 18), while 13% (n = 3) reported no change to their threshold and 9% (n = 2) were unsure. The oxygenation thresholds informing the decision to intubate varied between pre-COVID-19, early COVID-19, and present-day periods (Fig. 5).

#### Processes

Most respondents reported their intubation protocols changed multiple times during the COVID-19 pandemic (78%, n = 18). Respondents reported a shift from patient-individualized intubation processes prior to COVID-19 (96%, n = 22) to standardized algorithmic approaches early in the pandemic (61%, n = 14). A resurgence of patient-individualized processes occurred in the present-day period (74%, n = 17) (Appendix Fig. 5).

#### Occupational safety

The personal protective equipment (PPE) used for intubation of patients with suspected viral pneumonia increased notably during the early-COVID-19 period with sustained use in the present day (Appendix Fig. 6). Respondents reported increased use of negative-pressure airborne infection isolation rooms (AIIRs) for intubation (pre-COVID-19 (17%, n = 4) vs. early COVID (91%, n = 21), vs. present-day (83%, n = 19)). Respondents reported being very (65%, n = 15) or somewhat (35%, n = 8) concerned about becoming infected with COVID-19 during the intubation procedure. They rated their current intubation practices as being less (91%, n = 21) or equally likely (9%, n = 2) to result in transmission of communicable respiratory infections to themselves or their teams compared to their pre-COVID-19 practices.

#### Effectiveness & patient safety

Respondents described their current departmental intubation practices as being more (35%, n = 8), equally (61%, n = 14), or less likely (4%, n = 1) to result in first-pass success compared to pre-COVID-19 practices. Specifically, they rated using both VL and rapid sequence intubation (RSI) with paralysis as more or equally likely to facilitate first-pass success (100%, n = 23 for both). Regarding safety events, respondents reported their current intubation practices were more (13%, n = 3), equally (65%, n = 15), or less likely (22%, n = 5) to result in hypoxemia or hypotension for patients compared to their pre-COVID-19 practices.

#### Quality assurance

Respondents reported increased use of several quality assurance system factors in response to the pandemic, including pre-intubation checklists (48%, n = 11 vs. 78%, n = 18), electronic systems to summarize best-practices (0%, n = 0 vs. 22%, n = 5), in situ simulation training programs (57%, n = 13 vs. 70%, n = 16), visual posters and infographics summarizing best practices (10%, n = 2 vs. 60%, n = 12) and ongoing intubation-specific quality improvement programs (35%, n = 8 vs. 52%, n = 12).

#### Barriers & facilitators

Respondents identified barriers and facilitators to implementing novel airway management practices during the pandemic (Table 2).

**Table 2:** Barriers & facilitators to implementation of novel airway management practices

	Responses* (N = 23)
*Barriers, n (%)*	
Lack of group consensus as to the best approach	10 (43)
Physical supplies not organized adequately for use during intubation	6 (26)
Lack of a single institutional authority on intubation	6 (26)
Lack of adequate dissemination of revised airway practices	5 (22)
Physical supplies not available	4 (17)
*Facilitators, n (%)*	
A local clinical simulation program	16 (70)
A local quality improvement program	11 (48)
Access to free open-access content on new intubation protocols	11 (48)
Departmental presence of a knowledge translation specialist	9 (39)
An electronic knowledge translation tool (i.e., wiki, blog)	2 (9)

*Respondents were asked to select all that apply, columns will not sum to 100%

## Discussion

### Interpretation of findings

This survey exploring intubation practices in the pre-COVID-19, early-COVID-19, and present-day periods found that ED intubation practices were modified in response to the COVID-19 pandemic in every ED surveyed across Canada. This is the first published comparison of pre-COVID-19, early-COVID-19, and present-day intubation procedures across Canada. The extant literature describes several intubation practices utilized during the early-COVID-19 period, including intubation teams, standardized checklists, levels of PPE, primary use of RSI, intubation by the most experienced practitioner, early intubation, primary use of VL, use of a bougie, preference for supraglottic airways to ventilate pre-intubation, and the use of an “intubating box” [2–10, 22, 23]. This survey adds to the literature by characterizing the actual implementation of these recommendations in EDs across Canada. Many of these suggested modifications were reported by our respondents and supported by pre-COVID literature to optimize emergent intubation. Interestingly we noted temporal trends where some elements of the intubation process were adopted in the early-COVID-19 period with a subsequent return to baseline practice in the present-day, despite ongoing endemic SARS-CoV-2. By characterizing the changes made to ED intubation practices throughout the pandemic, noting which elements remain in use vs. which were abandoned, and understanding EM physician opinions and experiences regarding these factors, we will be able to recognize the continuing legacy of the COVID-19 pandemic on intubation practices and make informed decisions on which intubation process elements we prioritize.

### Previous studies

#### Team structures, simulation

Intubation-related adverse events are associated with a lack of a systematic approach [24]. Airway teams and in situ simulation have been suggested in pre-COVID intubation guidelines to decrease these adverse events [24–26]. Unsurprisingly, guidelines and observational studies published during the pandemic encouraged a structured and systematic process [27–29].

Pre-COVID-19 intubation literature supports operator experience as a factor that optimizes first-pass success [30, 31]. This prioritization of first-pass success was evidenced by respondent reports of exclusion of junior trainees from early-COVID-19 intubations in our sample. Exclusion from clinical duties, albeit for safety purposes, was eventually recognized as negatively impacting medical education [32]. Our evolving understanding of the risk of occupational exposure to SARS-CoV-2 and increasing access to vaccination contributed to the re-involvement of medical trainees to pre-COVID-19 levels in the present-day.

#### Equipment & medications

We found occupational infection with SARS-CoV-2 during aerosol-generating medical procedures (AGMPs) was a concern for many Canadian emergency physicians during the pandemic. Data collected during the SARS pandemic illustrated the dangers of AGMPs to healthcare workers (HCWs) and the importance of increased PPE. VL and RSI have been shown to improve and increase first-pass success rates [33–38] and potentially decrease occupational exposure to aerosolized SARS-CoV-2. This evidence informed many COVID-19 intubation algorithms, including methods of pre-oxygenation, rescue oxygenation, intubation techniques, respiratory support [2, 5, 7, 22, 39, 40], and likely many of the changes in equipment reported by our respondents during the early-COVID-19 period. Present-day has not seen a complete return to pre-COVID-19 oxygenation strategies. The continued use of VL, however, is interesting. Pre-pandemic debate about VL’s superiority to DL existed [41]. The continued use of VL in the present day may be related to use by previous non-adopters and the increased availability of the technology following the pandemic. Our reported findings of persistent airborne PPE, impermeable gowns, and eye protection use represent a positive cultural change for HCW safety.

#### Effectiveness & patient safety

Although many of the intubation practice changes reported have supporting evidence from the pre-COVID-19 period, the rapid implementation of numerous changes with an added focus on HCW safety introduced new safety concerns for patients. Respondents’ perceptions of the effectiveness and safety of changes to intubation practices are supported by two Canadian Studies. A recently published study comparing first-pass success before and after implementing a COVID-protected RSI Protocol found increased rates of first-pass success and no increase in adverse events [42]. An observational study conducted at the same CCEDRRN sites sampled in this survey also observed high rates of first-pass success and low rates of adverse events overall in ED intubations during the COVID-19 pandemic. In that sample, higher rates of post-intubation hypoxia were noted for patients positive for SARS-CoV-2 compared to SARS-CoV-2 negative patients, although patients with SARS-CoV-2 presented with lower oxygen saturations at baseline [14].

### Strengths & limitations

Generalizability of our results is limited by our sample, which was primarily academic or tertiary sites. Experiences of EM physicians working in rural, northern, and remote settings are not captured. Further, the available resources, including personnel and equipment, described in included EDs may not have been available in smaller centers. Despite the limitations of the sample frame, we achieved an excellent response rate through evidence-based survey science techniques. Furthermore, this is the only published study to compare intubation practices in the pre-COVID-19, early-COVID-19, and present-day periods across Canada.

Survey responses may be affected by recall bias. This survey relied upon reporting past behaviors in the pre-COVID and early-COVID periods. Respondent reported practice was, however, consistent with the observed practices during the COVID-19 pandemic described in our observational study of CCEDRRN sites [14].

### Clinical implications

The reported ongoing use of enhanced airborne PPE, VL, standardized protocolized intubation strategies, and quality assurance processes, along with provider opinions that current intubation practices were equally or more likely to result in first-pass success and more likely to protect HCWs from occupational exposure to airborne pathogens, suggest that a new paradigm for intubation practice has been established in Canada.

## Conclusions

This study offers a novel depiction of intubation practices in response to the evolving COVID-19 pandemic across Canada. As a result of a parallel observational study at the same sites, we know these changes were associated with a low risk of adverse events overall. The reported similarities in the changes made in CCEDRRN sites across Canada are compelling findings and could help inform a consensus on safe and effective emergent intubation of persons with communicable respiratory infections in the future. The identified barriers and facilitators to adopting novel intubation protocols in real clinical practice settings can directly inform future process changes. Further work is needed to understand how the COVID-19 pandemic impacted intubation practices in rural and remote EDs in Canada or other jurisdictions.

### Electronic supplementary material

Below is the link to the electronic supplementary material.

**Supplementary Material 1**: CAMP COVID-19 Questionnaire

**Supplementary Material 2: Appendix Figure 1**: Q13 ? Medical trainee participation in intubation over time. **Appendix Figure 2**: Q14 ? Ad hoc vs. formal airway management teams over time. **Appendix Figure 3**: Variation in rescue oxygenation techniques during attempted intubation. **Appendix Figure 4**: Variation in induction agents over time. **Appendix Figure 5**: Physician Directed versus Algorithmic Intubation Processes. Appendix Figure 6: Personal protective equipment use during intubation, over time

## Data Availability

Raw data are not available except with permission from the corresponding author with appropriate ethics approvals in place.
