# Supplementary material for: Characterizing intubation practices in response to the COVID-19 pandemic: a survey of the Canadian COVID-19 Emergency Department Rapid Response Network (CCEDRRN) sites
Source: BMC Emerg Med. 2023 Nov 24;23:139. doi: 10.1186/s12873-023-00911-w (PMC10675858; doi:10.1186/s12873-023-00911-w)
Supplement: Supplementary file 1 — Supplementary Material 1: CAMP COVID-19 Questionnaire [file 12873_2023_911_MOESM1_ESM.docx]

**CAMP COVID-19 Questionnaire**

In your clinical practice where do you intubate patients who need urgent or emergent airway management?

- ED
- ICU
- Hospital Ward
- OR
- Other (please specify)

Have the intubation protocols used in your ED changed in RESPONSE TO the COVID-19 pandemic? (Before COVID-19 vs. During COVID-19)

- Yes
- No
- Unsure

Have the intubation protocols used in your ED changed DURING THE COURSE OF the COVID-19 pandemic? (Early vs. Later on during COVID-19)

- Yes
- No
- Unsure

Were you more likely to intubate patients ‘sooner’ (i.e. with lower oxygen requirement and/or work of breathing thresholds) earlier in the pandemic compared to your practice today?

- Yes
- No
- Unsure

| What oxygenation threshold did you most commonly use to decide to intubate during the COVID-19 pandemic (all other factors being equal)? | | |
| --- | --- | --- |
| Prior to the pandemic | At the beginning of the pandemic | Currently |
| - 0-6 L supplemental O2 - 6-10 L supplemental O2 - 10-15 L supplemental O2 - >15 L supplemental O2 - High flow oxygen via nasal cannulae (e.g. Optiflow, Airvo)   - 20-39% FiO2   - 40-59% FiO2   - 60-79% FiO2   - 80-100% FiO2 - Non-invasive ventilation (i.e. BiPAP)   - 20-39% FiO2   - 40-59% FiO2   - 60-79% FiO2   - 80-100% FiO2 | - 0-6 L supplemental O2 - 6-10 L supplemental O2 - 10-15 L supplemental O2 - >15 L supplemental O2 - High flow oxygen via nasal cannulae (e.g. Optiflow, Airvo)   - 20-39% FiO2   - 40-59% FiO2   - 60-79% FiO2   - 80-100% FiO2 - Non-invasive ventilation (i.e. BiPAP)   - 20-39% FiO2   - 40-59% FiO2   - 60-79% FiO2   - 80-100% FiO2 | - 0-6 L supplemental O2 - 6-10 L supplemental O2 - 10-15 L supplemental O2 - >15 L supplemental O2 - High flow oxygen via nasal cannulae (e.g. Optiflow, Airvo)   - 20-39% FiO2   - 40-59% FiO2   - 60-79% FiO2   - 80-100% FiO2 - Non-invasive ventilation (i.e. BiPAP)   - 20-39% FiO2   - 40-59% FiO2   - 60-79% FiO2   - 80-100% FiO2 |

What guided this change in intubation threshold?

- - - Local working group
    - Primary literature
    - Medical Association Specialty Guidelines
    - Individual provider decision-making
    - Depletion of resources
    - Other (Please specify):

In your experience, does the need to transport patients with suspected or confirmed COVID-19 out of your facility to another facility for admission, lower your threshold for intubation?

- Yes
- No

Did you perform more awake and/or delayed sequence induction intubations as the pandemic progressed?

- Yes
- No
- Unsure

Select any of the following devices or tools you have ADOPTED during the course of the pandemic: (select all that apply)

- Routine LMA use
- Intubation boxes/bags
- Other (please specify)

Select any of the following devices or tools you have ABANDONED during the course of the pandemic:

- Routine LMA use
- Intubation boxes
- Other (please specify)

Who was/is most likely to perform intubations in your ED? (select one)

| Pre-COVID-19 | During COVID-19 |
| --- | --- |
| - Emergency Medicine Physician - Anesthesiology Physician - Critical Care Medicine Physician - Emergency Medicine Resident - Off-Service Resident - Other (please specify) | - Emergency Medicine Physician - Anesthesiology Physician - Critical Care Medicine Physician - Emergency Medicine Resident - Off-Service Resident - Other (please specify) |

Who were/are the members of your regular intubating (aka airway management) team in your ED? (select all that apply)

| Pre-COVID-19 | During COVID-19 |
| --- | --- |
| - Intubating staff physician - Physician trainees (resident physicians, medical students) - Nurse(s) - Respiratory Therapist(s) - Paramedic(s) - Other (please specify) | - Intubating staff physician - Physician trainees (resident physicians, medical students) - Nurse(s) - Respiratory Therapist(s) - Paramedic(s) - Other (please specify) |

Who was/is allowed to perform intubations in your ED? (select all that apply)

| Pre-COVID-19 | During COVID-19 |
| --- | --- |
| - Staff physicians only - Senior resident physicians - Junior resident physicians - Medical students - Other (please specify) | - Staff physicians only - Senior resident physicians - Junior resident physicians - Medical students - Other (please specify) |

Select the statement that most accurately applies to your ED:

| Pre-COVID-19 | During COVID-19 |
| --- | --- |
| - Intubations were performed ad hoc by staff working in the ED - Intubations were performed by a dedicated ED team (only some ED physicians assigned) - Intubations were performed by a dedicated hospital-wide airway response team - Other (please specify) | - Intubations were performed ad hoc by staff working in the ED - Intubations were performed by a dedicated ED team (only some ED physicians assigned) - Intubations were performed by a dedicated hospital-wide airway response team - Other (please specify) |

When selecting elements of your intubation strategy (i.e. induction agents, paralytics, laryngoscopy technique, etc.) do you most commonly use a physician-directed strategy (where you select and modify your approach to individualize for the patient) or a protocolized/algorithmic approach?

| Pre-COVID-19 | During COVID-19 |
| --- | --- |
| - Physician-directed - Protocolized/algorithmic | - Physician-directed - Protocolized/algorithmic |

Which of the following did/do you routinely use for pre-oxygenation prior to induction (select all that apply):

| Pre-COVID-19 | During COVID-19 |
| --- | --- |
| - Bag-mask ventilation (hand-bagging) - Bag-mask with a PEEP valve (no ventilation) - Non-invasive ventilation (i.e. BiPAP) - High flow oxygen via nasal cannulae (i.e. Optiflow) - Face mask (non-rebreather, venturi, etc.) - Nasal prongs - Other (please specify) | - Bag-mask ventilation (hand-bagging) - Bag-mask with a PEEP valve (no ventilation) - Non-invasive ventilation (i.e. BiPAP) - High flow oxygen via nasal cannulae (i.e. Optiflow) - Face mask (non-rebreather, venturi, etc.) - Nasal prongs - Other (please specify) |

How did/do you confirm endotracheal tube placement? (select all that apply)

| Pre-COVID-19 | During COVID-19 |
| --- | --- |
| - Qualitative end tidal carbon dioxide - Quantitative end tidal carbon dioxide - Auscultation for bilateral air entry - Direct visualization passing through the cords - Chest xray - Point of care ultrasound - Other (please specify) | - Qualitative end tidal carbon dioxide - Quantitative end tidal carbon dioxide - Auscultation for bilateral air entry - Direct visualization passing through the cords - Chest xray - Point of care ultrasound - Other (please specify) |

What personal protective equipment (PPE) did/do you use for intubation of patients with suspected or confirmed viral pneumonia? (select all that apply)

| Pre-COVID-19 | During COVID-19 |
| --- | --- |
| - Surgical face mask - N-95 face mask - P100 - Powered air-purifying respirator (PAPR) - Other respirator - Eye protection - Head protection - Impermeable gown - Gloves | - Surgical face mask - N-95 face mask - P100 - Powered air-purifying respirator (PAPR) - Other respirator - Eye protection - Head protection - Impermeable gown - Gloves |

What were/are your most commonly used induction medications to facilitate intubation? (select all that apply)

| Pre-COVID-19 | During COVID-19 |
| --- | --- |
| - Ketamine - Etomidate - Propofol - Midazolam - Fentanyl - Other (please specify) | - Ketamine - Etomidate - Propofol - Midazolam - Fentanyl - Other (please specify) |

What were/are your most commonly used paralytics to facilitate intubation? (select all that apply)

| Pre-COVID-19 | During COVID-19 |
| --- | --- |
| - I would normally not use a paralytic to facilitate intubation - Non-depolarizing neuromuscular blocker (i.e. rocuronium) - Depolarizing neuromuscular blocker (i.e. succinylcholine) - Other (please specify) | - I would normally not use a paralytic to facilitate intubation - Non-depolarizing neuromuscular blocker (i.e. rocuronium) - Depolarizing neuromuscular blocker (i.e. succinylcholine) - Other (please specify) |

What was/is your most commonly used device to facilitate intubation? (select all that apply)

| Pre-COVID-19 | During COVID-19 |
| --- | --- |
| - Direct laryngoscope - Video laryngoscope - Fibreoptic scope - Bougie - Other (please specify) | - Direct laryngoscope - Video laryngoscope - Fibreoptic scope - Bougie - Other (please specify) |

Where did/do you perform intubations in your ED for patients with suspected or confirmed viral pneumonias? (select all that apply)

| Pre-COVID-19 | During COVID-19 |
| --- | --- |
| - In an open space - In a closed-door space - In an airborne infection isolation room (AIIR) with negative pressure | - In an open space - In a closed door space - In an airborne infection isolation room (AIIR) with negative pressure |

In the event of a failed intubation attempt where your patient desaturates what techniques did/would you commonly use to improve oxygenation? (select all that apply)

| Pre-COVID-19 | During COVID-19 |
| --- | --- |
| - Bag-mask ventilation - Passive oxygenation with PEEP valve - Oropharyngeal airway - Supraglottic airway (e.g, Combitube, KingLT) - Other (please specify) | - Bag-mask ventilation - Passive oxygenation with PEEP valve - Oropharyngal airway - Supraglottic airway (e.g, Combitube, KingLT) - Other (please specify) |

Which of the following were/are routine elements of your airway management practice context? (select all that apply)

| Pre-COVID-19 | During COVID-19 |
| --- | --- |
| - In situ simulation training - A pre-intubation checklist - Visual Poster or infographic summarizing best practices - Electronic system to summarize best practices (i.e. a wiki or blog) - Ongoing quality improvement program specific to airway management | - In situ simulation training - A pre-intubation checklist - Visual Poster or infographic summarizing best practices - Electronic system to summarize best practices (i.e. a wiki or blog) - Ongoing quality improvement program specific to airway management |

In response to the COVID-19 pandemic many institutions have modified and standardized approaches to airway management, taking into account both patient and provider safety. How do you feel that your current COVID-19 airway practices compare to your pre-COVID-19 airway practices with respect to effectiveness?

- My current airway management practices are MORE LIKELY to result in first-pass success compared to my pre-COVID-19 practices
- My current airway management practices are EQUALLY LIKELY to result in first-pass success compared to my pre-COVID-19 practices
- My current airway management practices are LESS LIKELY to result in first-pass success compared to my pre-COVID-19 practices

How do you feel that your current COVID-19 airway practices compare to your pre-COVID-19 airway practices with respect to patient safety?

- My current airway management practices are MORE LIKELY to result in adverse events for patients (i.e. hypoxemia, hypotension) compared to my pre-COVID-19 practices
- My current airway management practices are EQUALLY LIKELY to result in adverse events for patients (i.e. hypoxemia, hypotension) compared to my pre-COVID-19 practices
- My current airway management practices are LESS LIKELY to result in adverse events for patients (i.e. hypoxemia, hypotension) compared to my pre-COVID-19 practices

How do you feel that your current COVID-19 airway practices compare to your pre-COVID-19 airway practices with respect to health care team safety?

- My current airway management practices are MORE LIKELY to result in transmission of communicable respiratory infections to myself or my team
- My current airway management practices are EQUALLY LIKELY to result in transmission of communicable respiratory infections to myself or my team
- My current airway management practices are LESS LIKELY to result in transmission of communicable respiratory infections to myself or my team

At the beginning of the COVID-19 pandemic, how concerned were you about being infected with COVID-19 during the intubation procedure?

- Very concerned
- Somewhat concerned
- Somewhat unconcerned
- Completely unconcerned

How would you rate each of the following components of airway management in terms of their impact on first-pass success during COVID-19-modified intubations?

Video laryngoscopy

- - - Use is MORE LIKELY to facilitate first pass success (as compared to direct laryngoscopy)
    - Use is EQUALLY LIKELY to facilitate first pass success (as compared to direct laryngoscopy)
    - Use is LESS LIKELY to facilitate first pass success (as compared to direct laryngoscopy)

Rapid Sequence Intubation (RSI) including paralysis

- - - Use is MORE LIKELY to facilitate first pass success (as compared to alternate methods with no paralysis)
    - Use is EQUALLY LIKELY to facilitate first pass success (as compared to alternate methods with no paralysis)
    - Use is LESS LIKELY to facilitate first pass success (as compared to alternate methods with no paralysis)

Which of the following have been barriers to the implementation of novel airway management practices during the COVID-19 pandemic in your ED? (select all that apply)

- Physical supplies not available
- Physical supplies not organized adequately for use during intubation
- Lack of group consensus as to the best approach
- Lack of a single identified institutional authority on airway practices
- Lack of adequate dissemination of revised airway practices
- Other (please specify)

Which of the following have facilitated the implementation of novel airway management practices during the COVID-19 pandemic in your ED? (select all that apply)

- Presence of a local quality improvement program
- Presence of a local simulation program
- Presence of an electronic knowledge translation tool
- Departmental presence of a knowledge translation specialist
- Access to FOAM-ED or social media articles on new intubation protocols
- Other (please specify)

Are there any components of an optimal emergency airway management system still missing from your clinical practice setting?

- Yes (please specify)
- No
- Unsure

Which clinical specialty do you practice? (select all that apply)

- Emergency Medicine
- Anesthesiology
- Critical Care Medicine
- Family Medicine
- Other (please specify)

How long have you been in clinical practice independently performing endotracheal intubation after completion of your clinical training?

- 0-5 years
- 6-10 years
- 11-15 years
- >15 years

Select your practice setting. (select all that apply)

- Academic/University centre
- Tertiary centre
- Community centre
- Other (please specify)

Does your center admit patients with suspected or confirmed COVID-19 who required intubation in the ED for ongoing mechanical ventilation?

- Yes
- No
- Unsure

Where do you primarily practice?

- Yukon
- Northwest Territories
- Nunavut
- British Columbia
- Alberta
- Saskatchewan
- Manitoba
- Ontario
- Quebec
- Nova Scotia
- New Brunswick
- Prince Edward Island
- Newfoundland and Labrador

How many patients with confirmed or suspected COVID-19 have you personally intubated since the beginning of the pandemic?

- 0
- 1-5
- 5-10
- 11-20
- 21-30
- >30
