# Supplementary material for: Characterizing intubation practices in response to the COVID-19 pandemic: a survey of the Canadian COVID-19 Emergency Department Rapid Response Network (CCEDRRN) sites
Source: BMC Emerg Med. 2023 Nov 24;23:139. doi: 10.1186/s12873-023-00911-w (PMC10675858; doi:10.1186/s12873-023-00911-w)

**Appendix**

Appendix Figure 1 – Q13 – Medical trainee participation in intubation over time


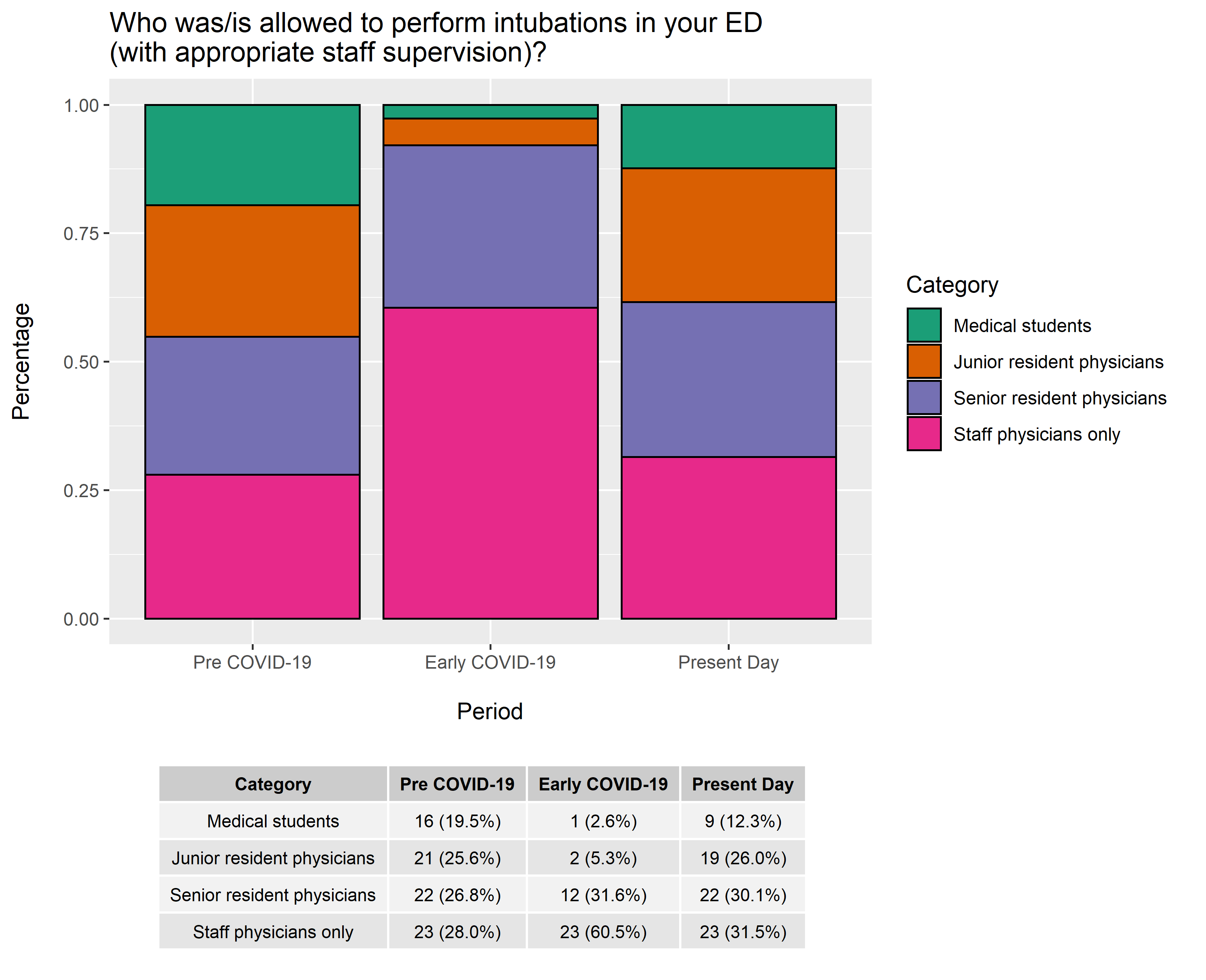


*Appendix Figure 2 – Q14 – Ad hoc vs. formal airway management teams over time*


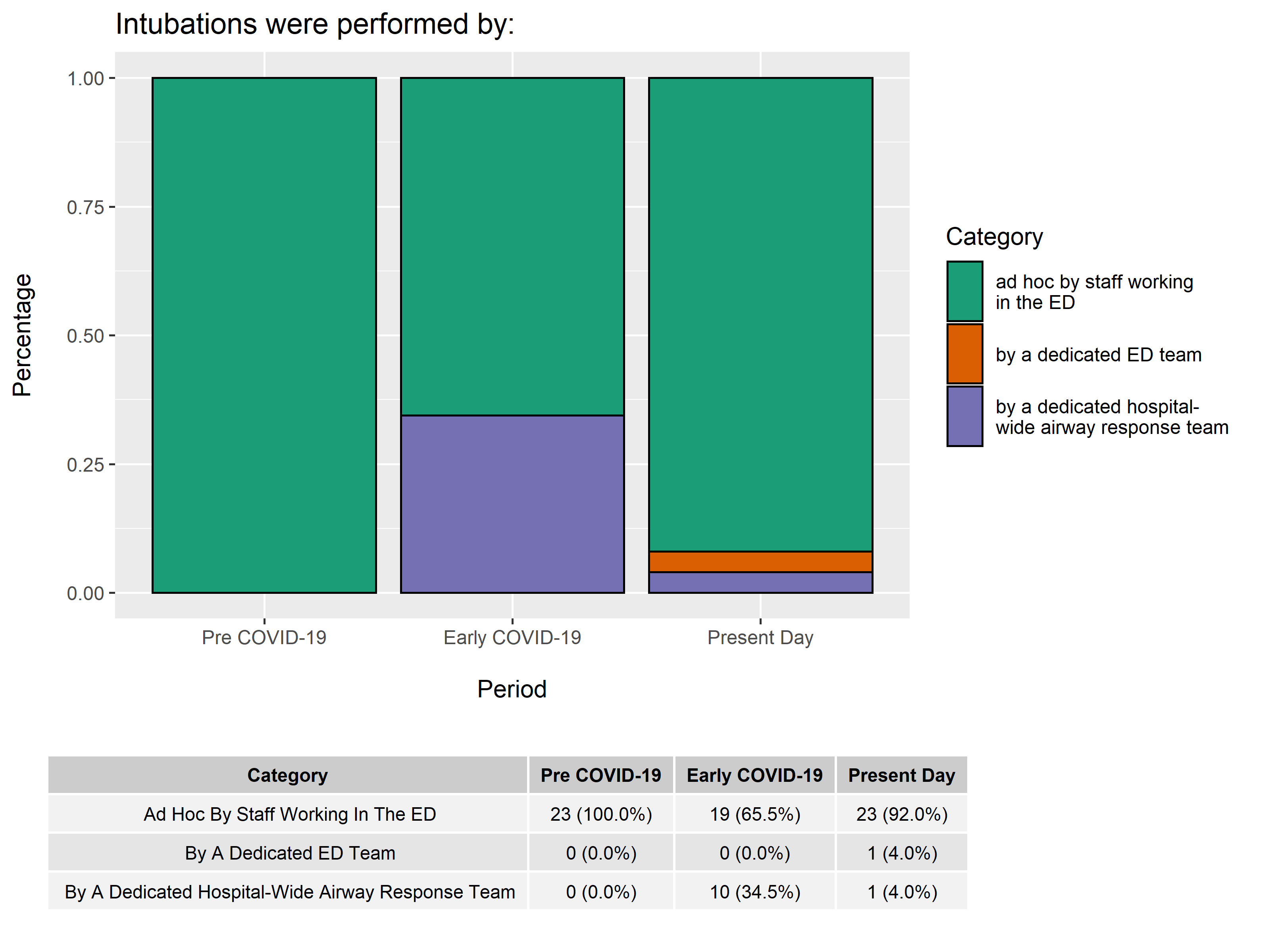


*Appendix Figure 3: Variation in rescue oxygenation techniques during attempted intubation*


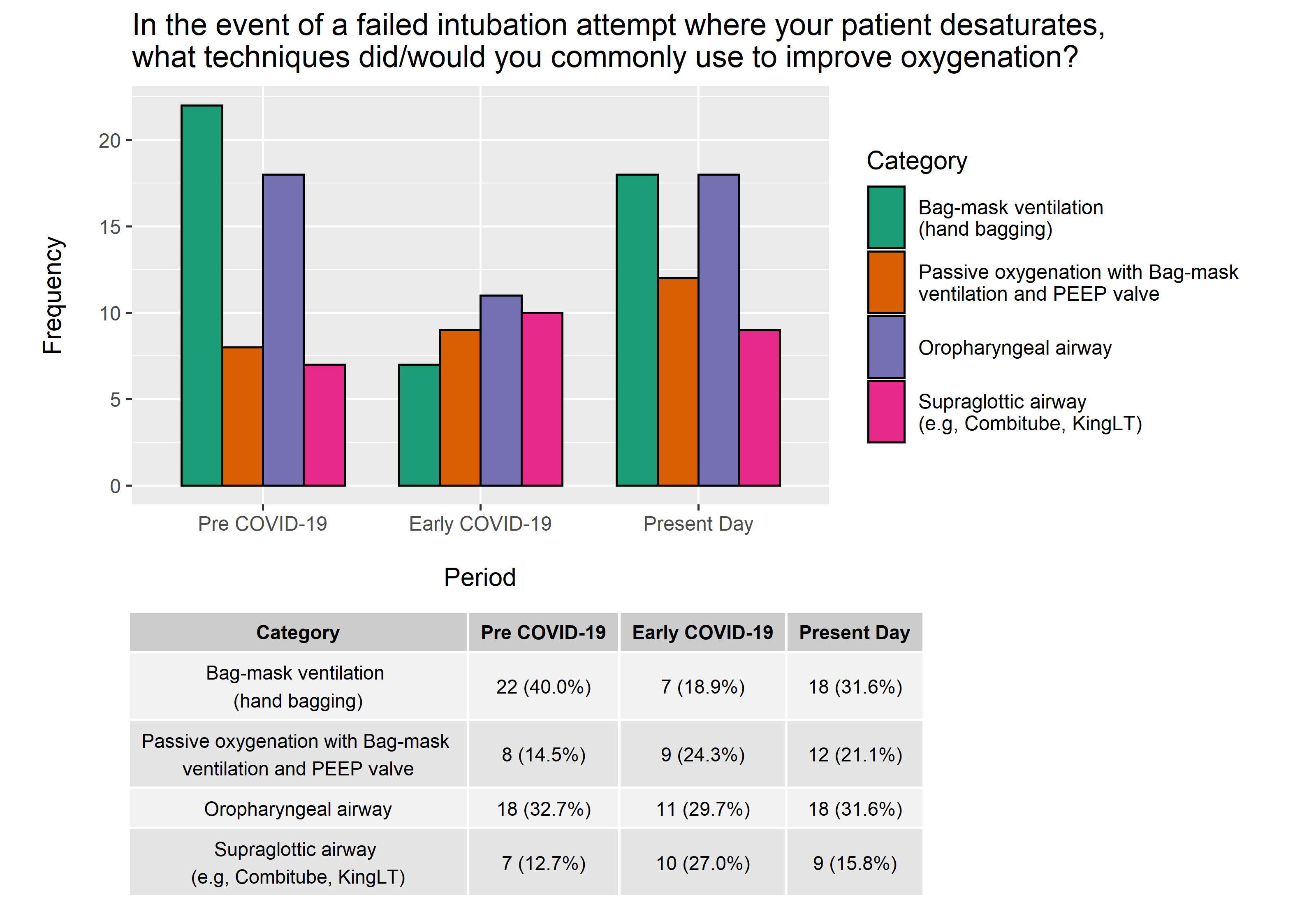


*Appendix Figure 4: Variation in induction agents over time*


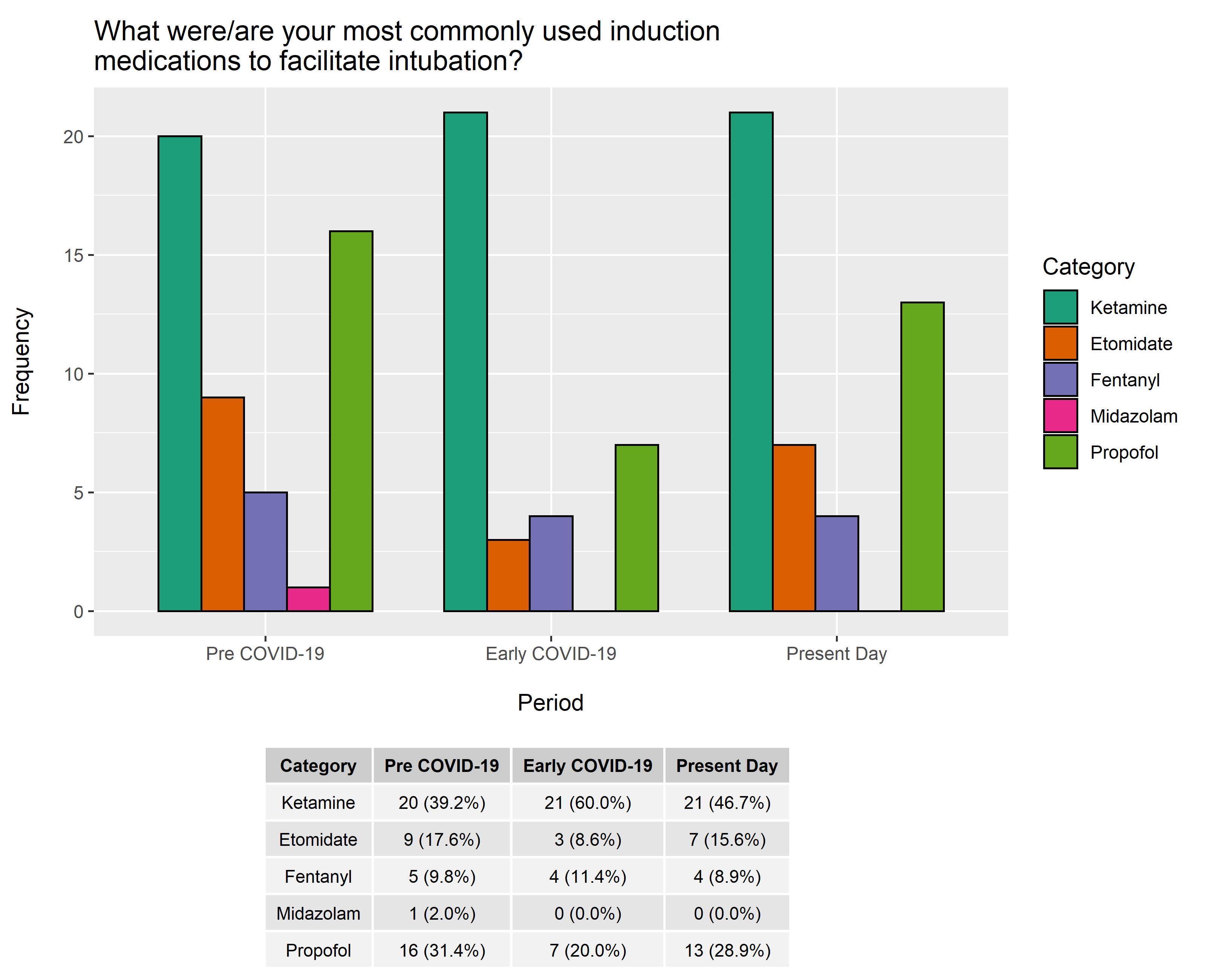


*Appendix Figure 5: Physician Directed versus Algorithmic Intubation Processes*


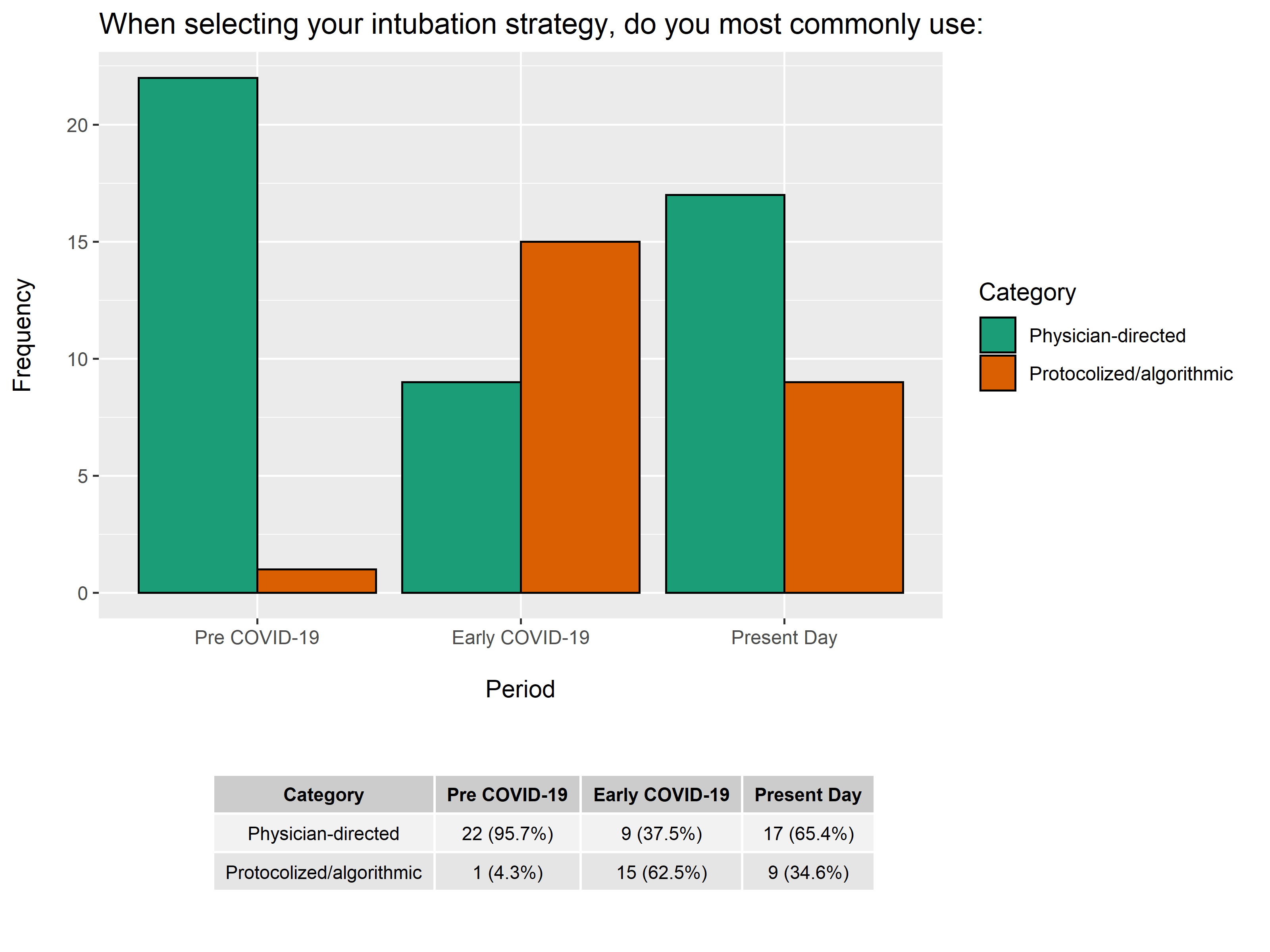


*Appendix Figure 6: Personal protective equipment use during intubation, over time*


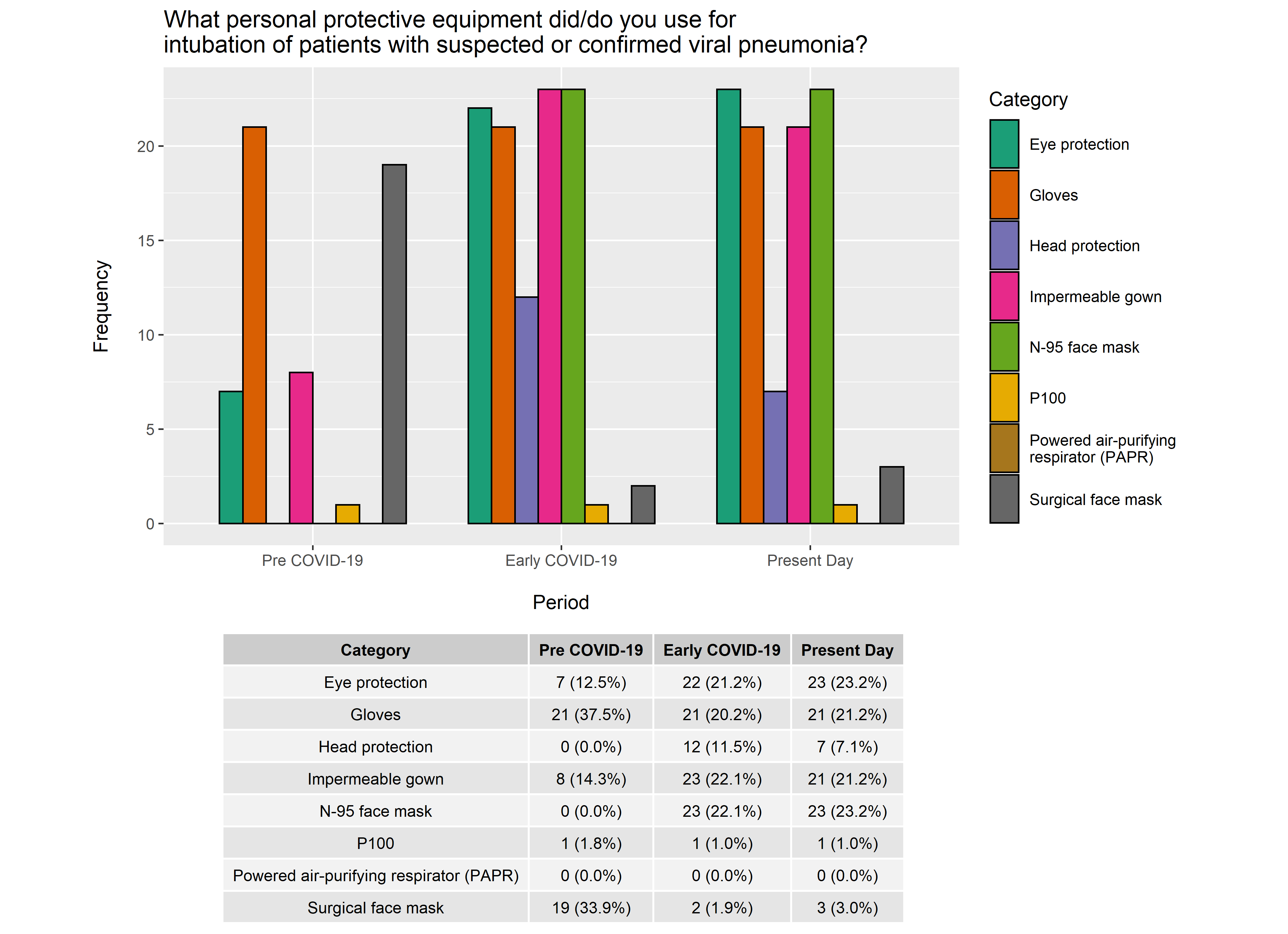

Supplement: Supplementary file 2 — Supplementary Material 2: Appendix Figure 1: Q13 ? Medical trainee participation in intubation over time. Appendix Figure 2: Q14 ? Ad hoc vs. formal airway management teams over time. Appendix Figure 3: Variation in rescue oxygenation techniques during attempted intubation. Appendix Figure 4: Variation in induction agents over time. Appendix Figure 5: Physician Directed versus Algorithmic Intubation Processes. Appendix Figure 6: Personal protective equipment use during intubation, over time [file 12873_2023_911_MOESM2_ESM.docx]
